# Propensity to intentional and unintentional mind-wandering differs in arousal and executive vigilance tasks

**DOI:** 10.1371/journal.pone.0258734

**Published:** 2021-10-19

**Authors:** Víctor Martínez-Pérez, Damián Baños, Almudena Andreu, Miriam Tortajada, Lucía B. Palmero, Guillermo Campoy, Luis J. Fuentes

**Affiliations:** Departamento de Psicología Básica y Metodología, Universidad of Murcia, Murcia, Spain; Julius-Maximilians-Universität Würzburg, GERMANY

## Abstract

We typically observe a decrement in vigilance with time-on-task, which favors the propensity for mind-wandering, i.e., the shifting of attention from the task at hand to task-unrelated thoughts. Here, we examined participants’ mind-wandering, either intentional or unintentional, while performing vigilance tasks that tap different components of vigilance. Intentional mind-wandering is expected mainly when the arousal component is involved, whereas unintentional mind-wandering is expected mainly in tasks involving the executive component. The Psychomotor Vigilance Task (PVT) assessed the arousal component, whereas the Sustained Attention to Response task (SART) assessed the executive component of vigilance. The two types of mind-wandering were probed throughout task execution. The results showed that the overall rate of mind-wandering was higher in the PVT than in the SART. Intentional mind-wandering was higher with the PVT than with the SART, whereas unintentional mind-wandering was higher with the SART than with the PVT. Regarding mind-wandering as a function of vigilance decrement with time-on-task, unintentional mind-wandering in the PVT increased between blocks 1 and 2 and then stabilized, whereas a progressive increase was observed in the SART. Regarding intentional mind-wandering, a progressive increase was only observed in the SART. The differential patterns of intentional and unintentional mind-wandering in both tasks suggest that, intentional mind wandering occurs mainly in arousal tasks in which propensity to mind-wander has little impact on task performance. However, unintentional mind-wandering occurs mainly in executive tasks as a result of a failure of cognitive control, which promotes attentional resources to be diverted toward mind-wandering. These results are discussed in the context of the resource-control model of mind-wandering.

## Introduction

Mind-wandering refers to the process during which our attention shifts from an ongoing task to thoughts that are not linked to task performance (for a review, see [1]). This phenomenon is thought to affect various situations in everyday life, as well as different domains of cognition, as evidenced when people perform cognitive tasks. Recently, it has been shown that task demands play a crucial role in the way mind-wandering occurs. In general, mind-wandering increases as task difficulty decreases [1], although the opposite pattern has also been observed [2]. Given the complex relationship between mind-wandering and task difficulty, some authors have drawn attention to the need of taking into account the type of mind-wandering that occurs during task performance, as well as the nature of the task at hand [3]. Mind-wandering can occur spontaneously (unintentional) or deliberately (intentional) [4], and task demands may differentially affect each type of mind-wandering. For example, Seli et al. [3] found that the rates of both intentional and unintentional mind-wandering were higher in the easy than in the difficult version of a working-memory load task, a result that is consistent with a large number of previous related studies. However, in an earlier study, Seli et al. [5] had observed just the opposite pattern, i.e., the rate of intentional mind-wandering was higher in the easy task than in the difficult task, and the other way around in the case of unintentional mind-wandering.

An inspection to the nature of the tasks may explain the divergent results of the two studies. While Seli et al. [3] used a cognitive task in line with most studies [6–8], Seli et al. [5] used two versions of the Sustained Attention to Response Task (SART). Importantly, in the easy version, the target digit appeared sequentially and, therefore, with high predictability. It is possible that predictability, rather than ease, may have been the cause of the atypical pattern of results observed in the study by Seli et al. [5].

The large repertory of cognitive tasks that have been used in the study of mind-wandering highlights the relevance of task difficulty. However, they say little about how mind-wandering changes as a function of time-on-task [9]. The ability to sustain attention for long periods of time plays an important role in a wide variety of activities in our daily lives, as well as in certain attention-demanding jobs. Particularly notable and pervasive are failures in task performance over time, a phenomenon known as the vigilance decrement [10–12]. Importantly, current theoretical accounts of the vigilance decrement have emphasized the involvement of mind-wandering in the decrease in performance over time-on-task. According to these theories, vigilance decrement would occur because the available attentional resources are shifted from the primary task to the mind-wandering process, which also consumes attentional resources [13–15]. The current model that best integrates and explains a wide variety of empirical findings related to the decrease in performance over time is the “resource-control” model of Thomson and colleagues [16]. The authors argued that the amount of resources available to perform a cognitive task are fixed, and the act of mind-wandering consumes those resources [14, 15]. In addition, mind-wandering is conceived as the default state, so that when individuals are faced with vigilance tasks, they exert executive control to avoid any deviation from the task at hand and keep the task goals in mind. The effectiveness of executive control is expected to decrease with time-on-task, which would result in participants devoting less and less resources to the primary task and more and more resources to mind-wandering, leading to the typical vigilance decrement function. Importantly, the empirical evidence, although still scarce, seems to support the model proposed by Thomson et al. [16]. Some relevant findings stem from studies that have assessed the effect of time-on-task on mind-wandering using a variety of cognitive tasks [17–21]. A common finding to the majority of studies is that a higher proportion of mind-wandering is accompanied by a decrease in performance towards the end of the task, in line with the predictions of the resource-control theory.

Although the rate of both intentional and unintentional mind-wandering has been considered to depend on task difficulty, the two types of mind-wandering have not been sufficiently explored in relation to the vigilance decrement phenomenon in vigilance tasks. This is particularly relevant because vigilance is not a unique entity (see [22] for a meta-analytic review), and two components have been theoretically dissociated [23]. Arousal vigilance occurs when people face monotonous and tedious tasks that require the maintenance of an optimal level of arousal to quickly respond to the upcoming target. The Psychomotor Vigilance Task (PVT [24]) is a prototypical task to assess the arousal component of vigilance. Executive vigilance, instead, occurs when the task goal is to detect infrequent stimuli and execute specific responses to them (e.g., the Mackworth Clock Test (MCT) [12]), or to retain responses to infrequent targets that are randomly presented (e.g., The Sustained Attention to Response Task (SART) [25]).

In the present study we used two vigilance tasks, the PVT and the SART, to assess the role of mind-wandering in performance decrement as a function of time-on-task. Although the two tasks usually show the typical vigilance decrement function, the processes involved differ between them. In the PVT, the decrement in performance is mainly due to the monotonous characteristic of the task, which leads to boredom and lack of motivation and may diminish participants’ interest, losing the focus on the task. In contrast, in the SART, it is thought to be due to a progressive failure of cognitive control to keep attentional resources available to inhibit responses to the target as time-on-task progresses.

In the present study, we set out to determine whether the two components of vigilance promote different rates of mind-wandering, and whether each type of mind-wandering is differentially affected as a function of decreasing vigilance with time-on-task, depending on the vigilance component involved. On the basis of previous research, we hypothesized that propensity to mind wander will be higher in the PVT than in the SART, as the former is a less demanding task than the latter, replicating the typical pattern of mind-wandering when researchers compared easy with difficult tasks [7, 8, 26]. When assessing the intentionality factor, we expect to find a higher rate of intentional mind-wandering with the PVT than with the SART, as the former requires less cognitive control than the latter. Conversely, we expect to find a higher proportion of unintentional mind-wandering with the SART than with the PVT, as we assume that this type of mind-wandering is mainly triggered by a failure in cognitive control. Finally, according to the resource-control model predictions [16], it is also reasonable to hypothesize that together with vigilance decrement, propensity to mind-wandering will increase across time-on-task in both tasks. Accordingly, we predict that the reported unintentional mind-wandering ratio towards the end of the task will be more prominent in the SART than in the PVT, due to the stronger cognitive control demands of the former task.

## Method

### Participants

We used G*Power software [27] to determine the sample size needed to have 80% power (1-β) to detect a medium effect size of *f* = 0.25 [28] at an alpha (α) level of .05, and given a correlation of ρ = .50 between repeated measures. This calculation suggested that a sample of 28 participants would be sufficient to detect even the medium effects sought. We recruited 34 participants without any physical, psychiatric or neurological illnesses in exchange of course credit, according to an incentive program approved by the Faculty of Psychology at the University of Murcia. We excluded the data of three participants who did not attend the second session of the experiment. We also removed the data of one participant who did not report any mind-wandering probe. The final sample consisted of 30 young adults aged 18–26 years (25 females, *M* age = 20.77, SD = 1.8). Prior to each experimental session, participants provided verbal consent. The study was conducted in accordance with the ethical standards laid down in the 1964 Declaration of Helsinki and was approved by the local ethics committee of the University of Murcia.

### Procedure and experimental tasks

In our within-participants design, all participants performed both the PVT and the SART in two different sessions separated by one week. Participants were randomly assigned to the order in which they performed the tasks, so that approximately half of the participants performed the SART in the first session and the PVT in the second session, and the other way around for the other half of the participants. The tasks were performed at a time interval from 10 am to 1 pm to prevent the performance from being influenced by participants’ chronotype and time of testing (see [29]).

Each task lasted 18 min. The PVT [24] was assumed to tap the arousal component of vigilance, whereas the SART [25] was assumed to tap the executive component of vigilance. In the PVT, each trial started with a blank screen during a random interval that lasted from 2 to 10 s. Suddenly, a red circle of 50 pixels diameter popped up at the center of the screen over a black background. Participants had to press the right button (number 5) of the Chronos device with the index finger of their dominant hand as quickly as possible when the red circle appeared. After responding, the screen went black and a new trial started.

In the SART, digits from 1 to 9 were presented in the center of the screen for 250 ms. Each digit was presented 100 times, adding a total of 900 stimuli. Digits appeared randomly in different font sizes (18, 27, 36, 45 and 54 point, Consolas). After each digit, a mask (a circle with a diagonal cross in the center) was displayed for 800 ms followed by a blank screen for 100 ms. The stimuli were presented in the center in white with a black background. Participants had to respond to each digit by pressing the right button of the response box (go trials) except for digit 3 (the target digit), in which case they had to refrain from responding (no-go trials). Participants were encouraged to give quick responses but to make as few mistakes as possible. At the beginning of the task, 18 practice trials were added, following the same procedure. In both tasks, we tested participants for mind-wandering using thought probes. The question “Which of the following responses best characterizes your state of mind just prior to the presentation of this display?” appeared on the screen with three response options: "On task”, "Intentionally mind-wandering", and " Unintentionally mind-wandering [5, 30]. Before starting, they were given verbal and written instructions both for answering the question and for the task itself. For the probe question, they were told that being on task meant that they were thinking about something related to the task (how hard it was, how boring it was, the buttons they had to press…). They were then provided with a definition of mind-wandering and briefly explained the difference between intentional and unintentional mind-wandering. Intentional mind-wandering refers to times when you voluntarily think about things unrelated to the task (e.g., the shopping list). Unintentional mind-wandering refers to times when you involuntarily think about things unrelated to the task (e.g., when a past event comes to mind). Participants were explicitly told that there were no right or wrong answers to the probes, but were encouraged to be honest in their responses. Participants were instructed to use the three buttons in the response box on the left (button 1, 2, and 3) to choose among the three response alternatives: on-task, intentional mind-wandering, or unintentional mind-wandering. In the PVT the probe appeared randomly in cycles of 8 trials, while in the SART it appeared randomly in cycles of 46 trials. Importantly, in both tasks the probe appeared randomly at ~50 second intervals.

During the tests, the Covid-19 protocol approved by the University of Murcia was strictly followed. The experimental tasks were performed on a 22-inch TFT monitor with a resolution of 1920 by 1080 pixels at a viewing distance of approximately 60 cm. Both tasks were programmed and analyzed using E-Prime-3 [31]. Responses were recorded with a 5-button Chronos device (Psychology Software Tools).

## Results

Data were analyzed with JASP 0.14.1 [32] adopting a significance level of α = .05. Supplementary data for this article can be found online at https://osf.io/yf5cm.

First, we attempted to replicate the vigilance decrement function that typically characterizes performance in vigilance tasks, regardless of the vigilance component involved. Next, we analyzed the overall mind-wandering in each task to test whether the rate of mind-wandering changes when either an arousal or an executive component is involved in the vigilance task. We repeated this analysis but now taking into account the type of mind-wandering involved, intentional or unintentional. Finally, we assessed the propensity for both types of mind-wandering across time-on-task for each vigilance task. This allowed us to determine how decreasing vigilance affects each type of mind-wandering as a function of the vigilance component involved.

### Vigilance decrement functions

The vigilance decrement in the PVT was analyzed through a one-way within-participants ANOVA with mean reaction times (RTs) as the dependent variable, and Block (1st to 4th) as the within-participants factor. The main effect of Block was significant *F*(3, 87) = 3.805, *p* = .013, *η^2^* = .116. Polynomial contrasts demonstrated that only the linear component was statistically significant, *t*(87) = 3.35, *p* < .001.

Similarly, for the analysis of the vigilance decrement in the SART, we performed a one-way within-participants ANOVA with accuracy (proportion of correctly inhibited responses to the target digit) as the dependent variable, and Block (1st to 4th) as the within-participants factor. The main effect of Block was significant *F*(3, 87) = 5.072, *p* = .003, *η^2^* = .149. Polynomial contrasts showed that only the linear component was statistically significant, *t*(87) = 3.37, *p* < .001.

These results illustrate the typical pattern of decrement in performance as a function of time-on-task that is characteristic of vigilance tasks, regardless of the vigilance component involved.

### Overall mind-wandering in the PVT and the SART

We conducted a paired samples *t-*test to assess the overall mind-wandering rate for each vigilance task (PVT, SART). Overall mind-wandering rates were obtained by the ratio of intentional plus unintentional mind-wandering responses to the total number of probes. The results indicated that there was a significant difference in overall mind-wandering rates between the two tasks, with higher reporting rates in the PVT (*M*_*PVT*_ = .677, *SD* = .20) than in the SART (*M*_*SART*_ = .483, *SD* = .17), *t*(29) = 4.59, *p* < .001, *d* = .839.

### Intentionality (intentional vs unintentional) of mind-wandering

We also examined the intentionality factor as a function of vigilance task by conducting a repeated-measures ANOVA with mind-wandering rate as the dependent variable, and Intentionality (Intentional, Unintentional) and Task (PVT, SART) as within-participant factors. Intentional and unintentional rates were calculated by dividing the relative proportion of each type of mind-wandering by the relative proportion of overall mind-wandering for each task. We found a main effect of Intentionality, *F* (1, 29) = 37.75, *p* < .001, *η^2^* = .36. The intentional mind-wandering rate was lower than the unintentional mind-wandering rate in both the PVT and the SART (PVT: *M*_*int*_ = .379, *SD* = .248; *M*_*unint*_ = .621, *SD* = .248, *t*(29) = 2.68, *p* = .006, *d* = .489; SART: *M*_*int*_ = .244, *SD* = .245; *M*_*unint*_ = .756, *SD* = .245, *t*(29) = 5.735, *p* < .001, *d* = 1.05). Importantly, the Intentionality × Task interaction was also significant, *F*(1, 29) = 4.186, *p* = .05, *η^2^* = .046. The analysis of the interaction showed that the intentional mind-wandering rate was significantly higher in the PVT (*M*_*PVT*_ = .379, *SD* = .248) than in the SART (*M*_*SART*_ = .236, *SD* = .245), *t*(29) = 2.05, *p* = .025, *d* = .374. In contrast, the rate of unintentional mind-wandering was significantly lower in the PVT (*M*_*PVT*_ = .621, *SD* = .248) than in the SART (*M*_*SART*_ = .756, *SD* = .245), *t*(29) = 2.05, *p* = .025, *d* = .374.

### Propensity to mind-wander across time-on-task

**Fig 1:**
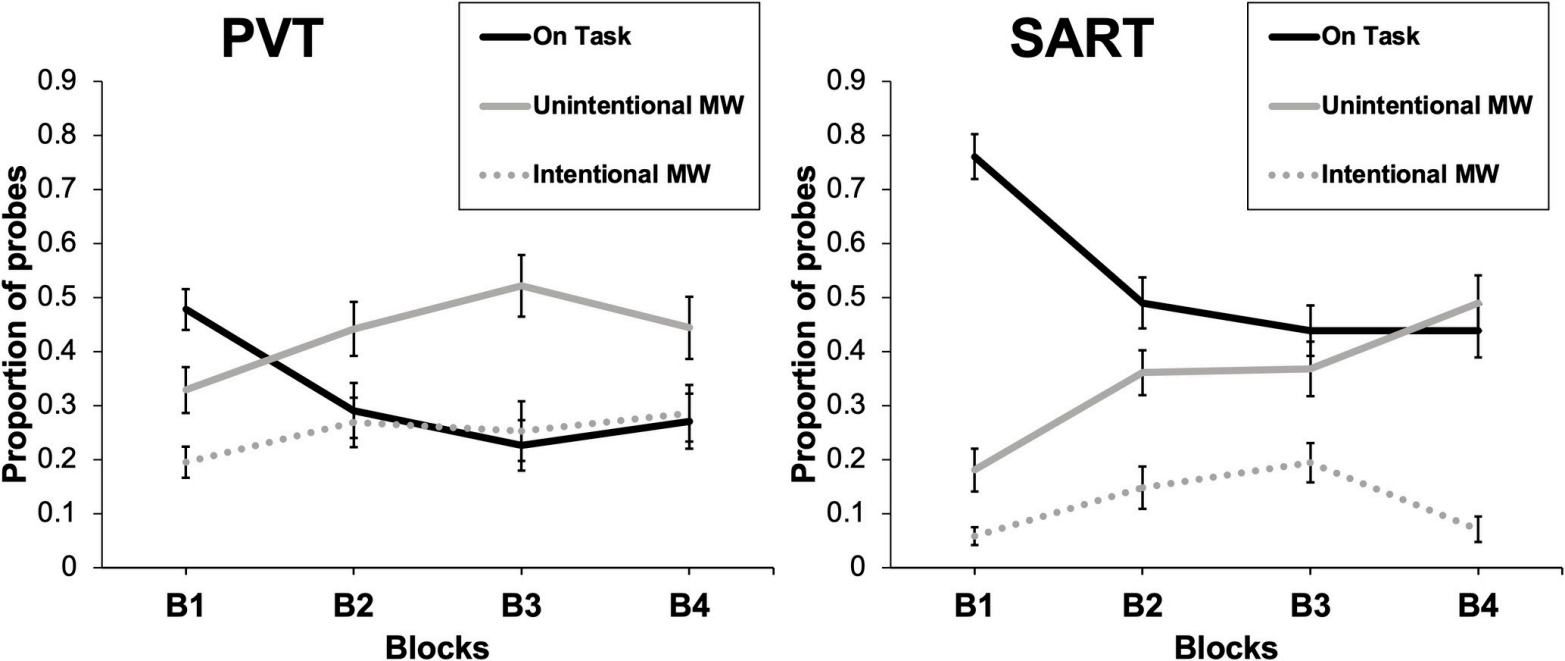
Mind-wandering across time-on-task. Proportion of both intentional and unintentional mind-wandering and on-task probes, as a function of block of trials.

To assess whether propensity to mind-wandering increases as performance decreases with time-on-task in the vigilance tasks, we conducted two 2 × 4 repeated-measures ANOVAs, one with the PVT and the other with the SART, with the rate of mind-wandering as the dependent variable, and Intentionality (Intentional, Unintentional) and Block (1st to 4th) as within-participant factors. The results are shown in Fig 1.

With the PVT, the main effect of Intentionality was significant, *F*(1, 29) = 5.78, *p* = .023, *η^2^* = .093. The intentional mind-wandering rate (*M*_*int*_ = .257, SD = .255) was lower than the unintentional mind-wandering rate (*M*_*unint*_ = .422, SD = .285). The effect of Block was also significant, *F*(3, 87) = 10.58, *p* < .001, *η^2^* = .034. Polynomial contrasts showed that both the linear and quadratic components were statistically significant (linear: *t*(87) = 4.34, *p* < .001; quadratic: *t*(87) = 3.59, *p* < .001). The quadratic trend showed that there was an increase in mind-wandering from B1 to B2, and a stabilization beyond B2. Accordingly, the effect of Block was not significant when Block 1 was removed from the analysis, *F* < 1. However, the trend in the mind-wandering rate was not modulated by the type of mind-wandering, as the Intentionality × Block interaction did not reach statistical significance, *F* < 1.

With the SART, the main effect of Intentionality was significant, *F*(1, 29) = 29.79, *p* < .001, *η^2^* = .239. As with the PVT, the intentional mind-wandering rate (*M*_*int*_ = .121, SD = .162) was lower than the unintentional mind-wandering rate (*M*_*unint*_ = .362, SD = .250). The main effect of Block was also significant, *F*(3, 87) = 18.19, *p* < .001, *η^2^* = .079. Importantly, the mind-wandering rate was modulated by the significant Intentionality × Block interaction, *F*(3, 87) = 6.12, *p* < .001, *η^2^* = .056. Polynomial contrasts showed that only the linear component was significant for unintentional mind-wandering, *t*(87) = 5.42, *p* < .001, whereas only the quadratic trend was significant for intentional mind-wandering, *t*(87) = 4.54, *p* < .001. The quadratic component was brought about by a decrement in intentional mind-wandering in the last block of trials.

## Discussion

The propensity to mind-wander is considered a pervasive phenomenon that usually occurs when people are required to perform certain activities for fairly long periods of time. However, previous studies have shown that the type of mind-wandering varies and may be modulated by task demands.

In the present study, we first assessed vigilance decrement functions in the two vigilance tasks, replicating the decrease in performance with time-on-task. We also observed that mind-wandering is more frequent with an easy task than with a task that demands cognitive control [3], and that unintentional mind-wandering is more frequent than intentional mind-wandering, regardless of the type of task being performed [33, 34]. While these previous findings refer to the effect of task difficult on the rate of mind-wandering, the novel contribution of the present research was twofold. First, we investigated the propensity to mind-wandering from a multicomponent conception of vigilance; and second, we assessed how the different types of mind-wandering may be affected by vigilance decrements as a function of the vigilance component involved. To achieve this goal, we chose a task thought to tap the arousal component of vigilance, the PVT, and a task thought to tap the executive component of vigilance, the SART.

In the PVT, participants need to achieve and maintain an adequate level of arousal to respond quickly to an infrequent target, so an arousal component is characteristic of this vigilance task. Little control is needed, as alternative responses are not required [25, 35]. In the SART, a great deal of cognitive control is needed to maintain a tonic level of alertness, as participants have to detect the presence of an infrequent target and, when the target is detected, inhibit the frequent response that was being emitted to non-target digits throughout the task. Thus, an executive component is characteristic of this vigilance task. Consistent with previous findings, both tasks showed the typical vigilance decrement function, but differed not only in the aforementioned mind-wandering pattern involved, but also in the time course of both types of mind-wandering as a function of time-on-task.

We found an opposite pattern of results according to the type of mind-wandering and type of task. While intentional mind-wandering was more frequent in the PVT than in the SART, unintentional mind-wandering followed the opposite pattern, being more frequent in the SART than in the PVT. This differential pattern of results suggests that both types of mind-wandering are caused by different processes.

Intentional mind-wandering can occur mainly when the task is monotonous, repetitive and boring, and it does not require cognitive control. Thus, participants can deliberately devote some attentional resources to their own thoughts at no cost to task performance. If intentional mind-wandering is mainly promoted in arousal-based vigilance tasks, we would predict that, in this type of tasks, intentional mind-wandering will be practically nonexistent when the individual is in circumstances that normally involve low levels of arousal. This is the case of sleep deprivation, or when the task is performed at the non-optimal time-of-day according to circadian rhythms [29]. In these situations, proper execution of the task will require greater cognitive effort, so it is to be expected that unintentional rather than intentional mind-wandering will be observed over time, as with tasks that require high levels of cognitive control. Further research should be conducted to assess this prediction.

In contrast, unintentional mind-wandering may occur primarily as a failure to maintain tonic alertness for longer periods, regardless of whether the task places low or high demands on cognitive control. This is supported by the increase in unintentional mind-wandering as performance decreases, regardless of task type. However, the present results also showed that such an increase in unintentional mind-wandering follows a different pattern in the PVT than in the SART. In the PVT, a task that requires little control, the steepest increase in mind-wandering occurs between blocks 1 and 2, and thereafter the mind-wandering rate stabilizes. In the SART, a task requiring high levels of cognitive control, we observed a progressive increase in unintentional mind-wandering as the task progresses. Taken together, the higher rate of unintentional mind-wandering in the highly demanding task, along with a progressive rather than abrupt increase in this type of mind-wandering as time with the task progresses, suggest that unintentional mind-wandering is best conceived as a failure to maintain attentional resources devoted to task performance.

The observed differences between the PVT and the SART in both overall and intentional/unintentional mind-wandering propensity could alternatively be explained by differences in the pacing rate of responding between the two tasks (see [36], for a demonstration of the effects of varying temporal contexts in arousal-based tasks). Whereas the PVT promotes a low response rate, the SART promotes a high response rate. However, it should be noted that differences between intentional and unintentional mind-wandering are also found in the study of Seli et al. [5] in which two versions of the SART were used, and consequently promoted a similar response rate. In our opinion, the differences in response rate between the PVT and the SART is a factor that adds to other task characteristics in triggering different types of vigilance. In other words, a low response rate may contribute to the task being monotonous, tedious, and lacking interest as it progresses, characteristics of the type of tasks that involve the arousal component of vigilance (e.g., the PVT). In contrast, a high response rate, as in the SART, may enhance cognitive control mechanisms to prevent continuous responses to non-target digits from affecting retention of the response to the target digit, which involves the executive component of vigilance. Thus, the difference in response rate between the two tasks can be considered a crucial factor promoting the involvement of different components of vigilance in each task.

The time course of mind-wandering as well as the vigilance decrement functions found here, allow us to test some predictions of the resource-control theory [16]. According to this account, the total resources available to cope with a vigilance task are fixed during the time course of the task, and individuals develop a strategic modulation in the allocation of resources between the primary task (external goals) and mind-wandering (internal goals). Furthermore, this model argues that it is not strictly a failure of executive control that causes mind-wandering to be observed from the outset. In fact, individuals may adjust the allocation of resources between mind-wandering and task goals in a way that maximizes off-task thoughts while preserving performance on the main task [8]. This is exactly what we observed in our pattern of probes when participants were performing the PVT. Because the executive demands of this task are quite low, participants devoted resources to mind-wander promptly and to a greater extent compared to when faced with an executive vigilance task, where the primary task is resource-intensive and it is not possible to initially devote many resources to mind-wandering. This model also proposes that mind-wandering is our default state, and that without the application of executive control our attentional resources tend to mind-wandering. The decrease in vigilance with time-on-task would appear as a consequence of executive control fading over time [37, 38], resulting in an insufficient allocation of attentional resources to the primary task. Consistent with this view, we found that participant’s propensity to unintentionally mind-wander, assumed to be explained by a failure of executive control, increases significantly in the last blocks of trials in the executive vigilance task (the SART) but not in the arousal vigilance task (the PVT).

To conclude, the results of the present study suggest that the resource-control theory [16] can be extended to account for the pattern and type of mind-wandering to be expected when people perform vigilance tasks, and that differ both in the demands on cognitive control and in the type of vigilance component involved.
